# Gender Differences in COVID-19 Deceased Cases in Jahrom City, South of Iran

**DOI:** 10.30476/BEAT.2021.89206

**Published:** 2021-04

**Authors:** Fatemeh Rahmanian, Naser Hatami, Marzieh Haghbeen, Rahim Raoufi, Ali Reza Abbasi, Heshmatollah Shakeri, Poyan Keshavarz, Elham Rafie, Mehdi Chegin, Esmaeal Raeyat Doost, Samaneh Abiri, Navid Kalani, Mahdi Foroughian, Mohsen Ebrahimi

**Affiliations:** 1 *Department of Emergency Medicine, Jahrom University of Medical Sciences, Jahrom, Iran*; 2 *Student Research Committee, Jahrom University of Medical Sciences, Jahrom, Iran*; 3 *Women’s Health and Disease Research Center, Jahrom University of Medical Sciences, Jahrom, Iran*; 4 *Department of Infectious Disease, Jahrom University of Medical Sciences, Jahrom, Iran*; 5 *Research Center for Social Determinants of Health, Jahrom University of Medical Sciences, Jahrom, Iran*; 6 *Department of Emergency Medicine, Faculty of Medicine, Mashhad University of Medical sciences, Mashhad, Iran*

**Keywords:** Blood pressure, COVID-19, Hyperlipidemia, Sex, Temperature

## Abstract

**Objective:**

To evaluate the clinical and epidemiological features of deceased patients and comparing the discrepancies between male and female patients based on high prevalence of coronavirus disease 2019 (COVID-19), its irreversible effects and the rising ‎mortality rate in Jahrom city,

**Methods:**

This is a descriptive-analytical retrospective study that was conducted from the beginning of March 2020 to the end of November 2020. The study population were included all patients with COVID-19 who admitted to Peymaniyeh Hospital in Jahrom and died of COVID-19. Clinical and demographic data were collected from medical records and analyzed by SPSS software.

**Results:**

In this study, 61 patients (57.54%) were men and 45 patients (42.36%) were women. The mean age was 68.7±18.33 in men and 68.82±14.24 in women. The mean hospitalization length was 9.69±7.75 days in men and 9.69±7.75 days in women patients. There was no statistically significant difference between men and women patients (*p*>0.05). The results showed that 17 (27.87%) men and 28 (45.9%) of women patients had hypertension and the prevalence of this disease was significantly higher in women than men (*p*=0.01). In this study, 7 (11.48%) men and 13 (21.31%) women had hyperlipidemia. The frequency of hyperlipidemia in women cases was significantly higher than in men patients (*p*=0.024). Men cases’ diastolic blood pressure (mean=77.53) was significantly higher than women’s diastolic blood pressure at the same time with a mean of 71.42 (*p*<0.05).

**Conclusion:**

The findings of the study represented the mortality rate in men which is higher than women patients. The prevalence of underlying diseases such as hypertension and hyperlipidemia were higher in women than men. Despite higher mortality among women, symptoms such as fever and dyspnea were less common in women than men.

## Introduction

Severe Acute Respiratory Syndrome-Coronavirus-2 (SARS-CoV-2) is a zoonotic virus that causes COVID-19 infectious pneumonia which was declared as a global epidemic by the World Health Organization (WHO) on January 30, 2020 [1]. First cases of COVID-19 were reported in a city of China and now has become a widespread pandemic with outbreaks in more than 200 countries [2]. The spread of the coronavirus has plunged all the countries of the world into a great humanitarian crisis. Recognition of the corona epidemic began in the city of Qom, Iran on February 20, 2020 [3]. Both COVID-19 and severe acute respiratory syndrome (SARS) diseases were associated with a severe inflammatory response with various symptoms [4, 5]. The severe SARS-CoV-2 infection patient’s characteristics and clinical outcomes information have been published in many studies; however, there is still a need for closely observing of patient’s clinical characteristics to reduce mortality [6]. Severe cases of COVID-19 could lead to severe pneumonia, severe respiratory failure, and death from multiple organ failure; while in severe cases, the usual symptoms of respiratory infection may not be present [7]. The most common clinical symptoms were fever and cough in addition to other nonspecific symptoms such as dyspnea, headache, muscle aches, and fatigue. About 30% of cases became complicated and the mortality were approximately 1.4% [8]. This study purpose is to evaluate clinical and epidemiological characteristics of deceased patients in Jahrom and compared the differences between men and women patients due to the high prevalence of COVID-19 and its irreversible consequences as well as increasing rate of mortality in Jahrom city.

## Materials and Methods

This study was a descriptive-analytical retrospective that were conducted from the beginning of March 2020 to the end of November 2020. The study followed the ethical principles for medical research involving human subjects (Helsinki Declaration) and was confirmed by the ethical research committee of Jahrom University of medical sciences (IR.JUMS.REC.1398.114). The population of the study were all patients with COVID-19 admitted to Peymaniyeh Hospital in Jahrom and died of COVID-19. Inclusion criteria were those with COVID-19 by a PCR test of the nasal swab. Exclusion criteria were disinclination to study. The sampling method was simple available sampling. 

The study primary outcome was demographic, clinical, and paraclinical of patient’s information. Data collection was performed at the patient’s arrival at the hospital. The patient’s daily vital signs were recorded three times per day and its mean was calculated for each day. Finally, the first day’s vital signs (time 1), the middle day of hospitalization (time 2), and the final day (time 3) of hospitalization results were analyzed. 

Descriptive data were expressed as number (%) for the categorical data and mean ± SD for continuous data. Data analysis was conducted by using SPSS software version 21, descriptive statistical values, comparative statistical tests as Chi-Square for categorical data and t-test for continuous data. Statistical significance was considered at *P* <0.05.

## Results

We examined the patient’s clinical characteristics who died of COVID-19. Demographic characteristics such as age, gender, length of hospital stay, body mass index (BMI), underlying diseases and symptoms of these patients can be observed in Table 1. Sixty-one patients (57.54%) were men and 45 (42.36%) were women. The mean age was 68.7±18.33 and 68.82±14.24 in men and women, respectively. The mean body mass index in men and women was equal to 25.7±4.34 and 25.49±6.75, respectively. There was no statistically significant difference between men and women in this assess (*p*>0.05). The mean number of hospitalization days was 10.98±8.19 and 9.69±7.75 in men and women, respectively which was not statistically significant (*p*>0.05). Afterwards, we examined the type and prevalence of underlying diseases in the studied patients. The results of this study showed that 17 (27.87%) and 28(45.9%) of men and women had hypertension, respectively, and the prevalence of this disease in women was significantly higher than in men (*p*=0.01). The results showed that the most common underlying disease after hypertension were diabetes mellitus in patients. Twenty men patients (32.79) and 18 (29.51) of women had diabetes mellitus. Hyperlipidemia was also one of the diseases observed in this study population. Seven men patients (11.48%) and 13 (21.31%) of women had hyperlipidemia. The number of women with hyperlipidemia was significantly higher than in men as observed in the prevalence of hypertension disease (*p*=0.024). The prevalence of respiratory diseases was not remarkable among both men and women patients. In this procedure 5(8.2%) men and 3 (4.92%) of women patients had respiratory diseases. Four (6.56%) men and 6 (9.84%) of womens patients had kidney disease. Thyroid disorders were another underlying diseases that was less prevalent among men and women. Two (3.28%) and 3 (4.92%) of men and women in had hyperthyroidism, respectively. Finally, the overall results of the study showed that 1 (1.64%) of men and 3 (4.92%) of women patients had hypothyroidism. The most common symptoms of COVID-19 patients were dyspnea 40(65.57%), cough 34 (55.74%), and fever 30 (49.18 %) in men, respectively. The most common symptoms in women were dyspnea 29 (47.54%), cough 23 (37.7%), and fever 18 (29.51%); there was no statistically significant difference in the case of the symptoms between two groups (Table 1).

**Table 1:** Demographic information and the prevalence of symptoms in study patients

		**Male N=61**	**Femalen=45**	***p*** **-value**
Age, Years, Mean±SD		68.7±18.33	68.82±14.24	0.34
BMI, W/M^2^, Mean±SD		25.7±4.34	25.49±6.75	0.87
Hospitalization days, Day, Mean±SD		10.98±8.19	9.69±7.75	0.41
Mechanical ventilation		47(77.04)	36(80)	0.28
Underlying disease, n(%)	Hypertention	17(27.87)	28(45.9)	0.01*
	Kidney Disease	4(6.56)	6(9.84)	0.23
	Diabetes Melitus	20(32.79)	18(29.51)	0.44
	Hyperlipidemia	7(11.48)	13(21.31)	0.02*
	Respiratory disease	5(8.2)	3(4.92)	0.76
	Hyperthyroidism	2(3.28)	3(4.92)	0.41
	Hypothyroidism	1(1.64)	3(4.92)	0.17
Symptoms, n(%)	Fever	30(49.18)	18(29.51)	0.34
	Dyspnea	40(65.57)	29(47.54)	0.90
	Cough	34(55.74)	23(37.70)	0.63
	Chils	5(8.2)	6(9.84)	0.39
	Anorexia	14(22.95)	8(13.11)	0.51
	Diarhea	4(6.56)	5(8.20)	0.40
	Fatigue	24(39.34)	14(22.95)	0.38
	Nausea and Vomiting	9(14.75)	10(16.39)	0.32
	Sputum	4(6.56)	2(3.28)	0.64
	Chest pain	6(9.84)	3(4.92)	0.56
	Body pain	17(27.87)	9(14.75)	0.35
	Rash	1(1.64)	0(0)	0.38
	Headache	8(13.11)	3(4.92)	0.28
	Ageusia	6(9.84)	3(4.92)	0.56
	Anosmia	4(6.56)	2(3.28)	0.64

**Fig. 1:**
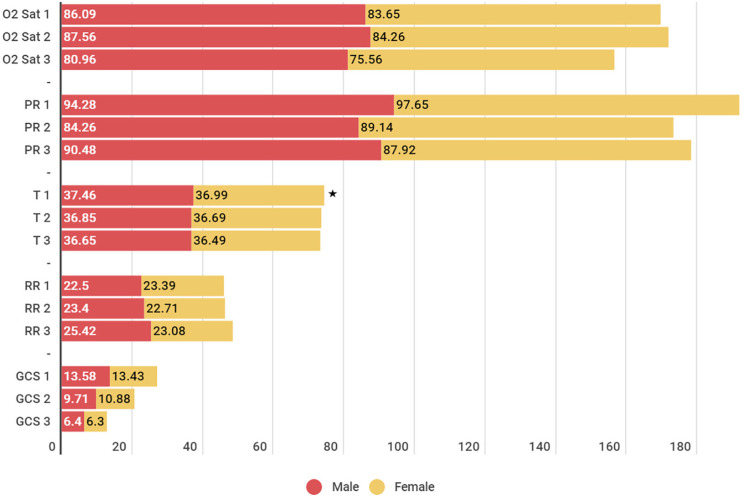
Evaluation of vital signs of patients. Statistically significant difference among genders

Figure 1 shows that there is a statistically significant difference between blood Oxigen saturation (O2sat), pulse rate (PR), respiratory rate (RR), and Glasgow Coma Scale (GCS) in 3 time periods. But the mean body temperature (T1) in men and women was 37.46 and 36.99, respectively, and T1 in men was significantly higher than in women (*p*<0.05).

**Fig. 2:**
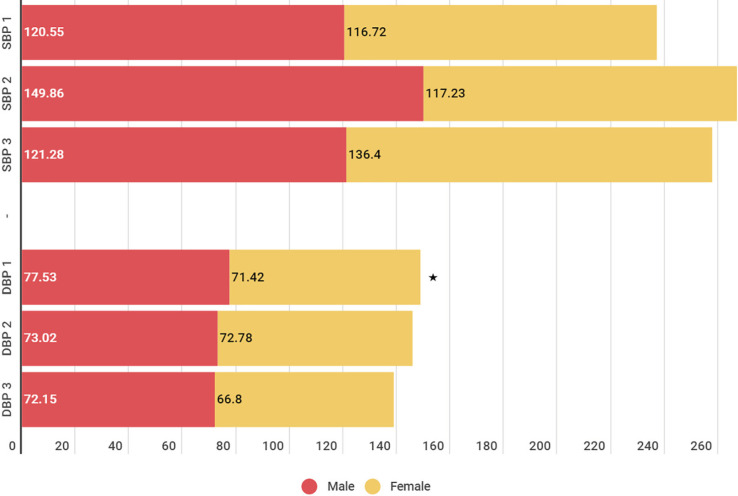
Blood pressure compression. Statistically significant difference among genders

The comparison of patients’ blood pressure can be observed in three times period (Figure 2). The results of the present study did not show any statistically significant differences in the three mentioned periods in systolic blood pressure (SBP). But men patient’s diastolic blood pressure (DBP) (mean=77.53) was significantly higher than mens at the same time with a mean of 71.42 (*p*<0.05) (Figure 2).

## Discussion

The present study was evaluated the COVID-19 clinical outcome differences among the genders. As this study cases were all the deceased patients’ data in Jahrom city till November 2020, we could say that men patients were died more than women of COVID-19 but we had a calculation limitation in the mortality rate due to no access of reliable epidemiologic data in all COVID-19 cases in this city. Chen *et al*., study showed that 41 (66%) of their patients were men and 21 (34%) were women and the mortality rate was higher in men than in women [9] which is in line with our study. In a study by Jin *et al*., [10] the number of men patients who died was significantly higher than in women (*p*=0.035) and the mortality rate in men was 2.4 times higher than women. Marcon *et al*., [11] found that older men were more resistant to COVID-19 than women (similar in age). In other words, the results of their study showed that the mortality rate in women is higher than men. Pérez-López *et al*., [12] examined the death rate due to COVID-19 in 23 European countries. The results of their study showed that the mortality rate in men is significantly higher than women. This was confirmed in our study; while the differences in mortality was not so much. In the case of the demographic and clinical outcomes in our study, there was no statistically significant difference in age among the genders. In the Jin *et al*. study, the mean age of the deceased women and men was 63 years and 59 years, respectively. Also, the prevalence of underlying diseases of hypertension and diabetes was higher in men than women. However, in present study, the prevalence of hypertension in deceased women was significantly higher than men (*p*=0.01).

Meng *et al*. examined 168 severe patients with COVID-19. 12.8% of men with COVID-19 died and the most common underlying diseases in both men and women were hypertension, diabetes, and cardiovascular diseases. There was no statistically significant difference between men and women in terms of the prevalence of underlying diseases [13]. However, in the present study, the prevalence of hypertension as an underlying disease was significantly higher in women than men (*p*=0.01). 

Jing Chen *et al*., [14] study found that complications such as acute respiratory distress syndrome, acute kidney injury, septic shock, heart damage, and coagulation disorders are less common in women. But in the present study, these factors’ occurrence was not recorded to be taken into account for further studies. 

A study by Qiu *et al*. examined a total of 2401 patients. 66.6% of COVID-19 patients were men with a mean age of 69.9 years. Common symptoms of the deceased cases were included fever (100%), shortness of breath (89.38%), cough (78.42%), and fatigue (22%). High blood pressure, chronic cardiovascular disease, diabetes, and chronic cerebrovascular disease were common among the deceased patients [15]. The most common symptoms in the present study were fever, cough, and dyspnea as well as Qiu *et al*., [15] study. In the study of Zhao *et al*., the most common symptoms in patients were cough (82.4%), fever (64.8%), fatigue (38.5%), and diarrhea (15.4%), respectively [16].

Zhang *et al*., study examined clinical data in 82 confirmed laboratory deaths. The majority of patients who died had comorbidities (76.8%) including hypertension (1.56%), heart disease (7.20%), diabetes (3.18%), cerebrovascular disease (2.12%), and cancer (3.7%) [17]. In the present study, the most common underlying diseases was diabetes mellitus‎ in in both genders. 

A significant point in the present study was that the prevalence of hyperlipidemia in 13 women (21.31%) deceased patients which was significantly higher than men with 7 (11.48%) hyperlipidemia prevalence (*p*=0.024). 

Our study showed that the difference between men and women in some vital signs (temperature and diastolic blood pressure) but this finding was not evaluated in other studies. 

Li *et al*., [18] studied 47 patients with COVID-19. The diabetes prevalence as an underlying disease was higher in women than men. Also in their study, 2 women had hyperthyroidism. The prevalence of hypertension in their study was higher in women than men and a woman had hyperlipidemia than in comparing with our study, we did not observe such differences except for hyperlipidemia. 

The main new finding of the present study was the possible relationship between hyperlipidemia and the COVID-19 mortality in women cases and also the differences in temperature vital signs and diastolic blood pressure between the deceased men and women cases. Therefore, we suggest for further studies to evaluate the lipid profile role in the COVID-19 mortality especially in women individuals.

## Limitations

This study is based on a retrospective comparison of only deceased patients that have been examined for core interest variables in resulting of some interest loss. The baseline comorbidities magnitude that could have played a part in the lethal result, cannot be determined; however, we could only examine gender variations between deceased individuals. The death sex-specified risk factors could be resolved by a comparison between survivors and no-survivors due to COVID-19. Also, these data were recorded at a different peaks of the COVID-19 outbreak in the city. Our clinical experiences remind us different pattern of disease presentation in patients’ arrival in each outbreak and may have biased the results. 
